# Aberrant alteration of peripheral B lymphocyte subsets in hepatocellular carcinoma patients

**DOI:** 10.7150/ijms.79305

**Published:** 2023-01-22

**Authors:** Jiaoxiang Wu, Yue Wang, Shihao Bai, Hanxiao Sun, Jie Zhang, Jie Shu, Yajie Wang, Meiyu Tan, Lida Zhou, Biao Huang, Qiuhui Pan, Huiming Sheng

**Affiliations:** 1 Department of Clinical Laboratory, Tongren Hospital, Shanghai Jiao Tong University School of Medicine, Shanghai, China.; 2 Key Laboratory for Translational Research and Innovative Therapeutics of Gastrointestinal Oncology, Hongqiao International Institute of Medicine, Tongren Hospital, Shanghai Jiao Tong University School of Medicine, Shanghai, China.; 3 Shanghai 10th people's hospital affiliated to Tong Ji University school of medicine, Shanghai, China.; 4 Key Laboratory of Systems Biomedicine (Ministry of Education), Shanghai Centre for Systems Biomedicine, Shanghai Jiao Tong University, Shanghai, China.; 5 Immunoassay Laboratory, College of Life Sciences and Medicine, Zhejiang Sci-Tech University, Hangzhou, China.; 6 Department of Clinical Laboratory Medicine, Shanghai Children's Medical Center, School of Medicine, Shanghai Jiao Tong University, Shanghai, China.

**Keywords:** Hepatocellular carcinoma, Interleukin-10-producing regulatory B cells, Marginal zone B cells, Follicular B cells, IL-10.

## Abstract

Although B lymphocytes are widely known to participate in the immune response, the conclusive roles of B lymphocyte subsets in the antitumor immune response have not yet been determined. Single-cell data from GEO datasets were first analyzed, and then a B cell flow cytometry panel was used to analyze the peripheral blood of 89 HCC patients and 33 healthy controls recruited to participate in our research. Patients with HCC had a higher frequency of B10 cells and a lower percentage of MZB cells than healthy controls. And the changes in B cell subsets might occur at an early stage. Moreover, the frequency of B10 cells decreased after surgery. Positively correlated with B10 cells, the elevated IL-10 level in HCC serum may be a new biomarker in HCC identification. For the first time, our results suggest that altered B cell subsets are associated with the development and prognosis of HCC. Increased B10 cell percentage and IL-10 in HCC patients suggest they might augment the development of liver tumors. Hence, B cell subsets and related cytokines may have predictive value in HCC patients and could be potential targets for immunotherapy in HCC.

## Introduction

Primary liver cancer is one of the most commonly diagnosed cancers with high mortality and morbidity 1. Hepatocellular carcinoma (HCC), characterized as a refractory malignancy, represents nearly 90% of primary liver cancer cases and affects 350 million people worldwide 2. Epidemiological studies show that the development of HCC is always accompanied by long-term chronic infection with hepatitis B virus (HBV) and hepatitis C virus (HCV) 2, 3. Surgical treatment and transarterial chemoembolization (TACE) are two means of treatment for HCC. However, the effectiveness is low, and HCC still exhibits fatal recurrence 4. Therefore, an increasing number of studies have been conducted to evaluate the immune response in the tumor microenvironment during the process of carcinogenesis 5. Great efforts have been made to identify effective immunotherapeutic approaches for HCC treatment by trying to harness the immune response to HCC 6 since immunotherapy is considered a relatively effective way to reverse the immunosuppressive tumor environment.

In recent years, the theory of tumor immune surveillance has been extensively accepted. The roles of lymphocytes and several cytokines in the antitumor immune response have been widely studied. Solid evidence shows that T cell subsets, such as regulatory T (Treg) cells, T helper 17 (Th17) cells, and CD8+ T cells, infiltrate liver tumors 7, 8. The presence of T cell subsets in human tumors has been explored. Treg cells are involved in HCC development, metastasis, progression, and recurrence. Many current literatures focusing on the opposite etiological function of Tregs in viral and autoimmune chronic liver disease in HCC evolution 9. Several publications are showing that the frequency of Treg cells in human peripheral blood is increased significantly in HCC patients and can be a prognostic factor in these patients 7.

In addition to the research on T cell subsets, increasing focus is being given to the roles of B cells in regulating and modulating the immune response 10. The known functions of B cells in the immune response include the production of antibodies against various antigens, such as viruses, bacteria, and tumor cells; presentation of antigens to T cells; secretion of cytokines; and expression of molecules that can act as costimulatory factors 10. Immunohistochemistry evidence demonstrates that B cell subsets infiltrate liver tumors and play a critical role in tumorigenesis in HCC patients and mouse models 11, 12. However, the precise identification and validation of proper B cell subsets as HCC biomarkers are not complete.

Both marginal zone B cells (MZB cells,) and follicular B cells (FoB cells) can participate positively in the immune response by stimulating CD4^+^ T cells and differentiating them into antibody-producing cells in different temporal and spatial windows 13, 14. Detection of MZB cells were by CD19^+^CD21^+^CD23^-^, and for FoB cells were by CD19^+^CD21^-^CD23^+^ 15. Human MZB cells share many properties with their mouse counterparts and circulate peripherally rather than in local areas in secondary lymphoid organs. This striking feature makes MZB cells a potential player in different clinical settings, including cancer and infection 16. In contrast to precursor cells, FoB cells are primarily enriched in splenic follicles 13. MZB cells act more rapidly than FoB cells when exposed to pathogens since multiple signals are integrated, leading to a lower activation threshold 14. Since the marginal zone has rich blood flow, MZB cells are exposed to blood circulation and can shuttle between the marginal zone and follicles. Compared with FoB cells, MZB cells are involved in the immune response at an earlier stage and higher levels 13.

Additionally, B10 cells, an important subset of regulatory B cells, can suppress the immune response by releasing suppressive cytokines, such as IL-10 and TGF-β, and can also suppress the T cell response via direct contact with pathogenic T cells 17. The frequency of B10 cells in the peripheral blood in autoimmune disease patients, measured by evaluating the surface molecules CD19, CD5, and CD1d, is relatively low 18. But the percentage of B10 cells increased in peripheral blood of ESCC tumor patients 19, and depletion of B10 cells in the syngeneic lymphoma model can dramatically enhance tumor clearance 20.

Although B cell subsets are surely present in the tumor-immune microenvironment, few studies have investigated the diagnostic and targeted therapeutic value of B cell subsets in HCC patients. Our research aims to explore the characteristics of peripheral B cell subsets in HCC patients. The results will help identify specific targets for HCC patient treatment and provide guidance for further exploration of the functions and mechanisms of B cell subsets in HCC patients.

## Materials and Methods

### Ethics statement

Our research was approved by the Ethics Committee of Shanghai Tenth People's Hospital (Approval number: RES-2016-14). Informed consent was obtained from each participant.

### Public single-cell RNA-seq data analysis

We used two public healthy PBMC datasets from 10X Genomics (https://www.10xgenomics.com) and TISCH (http://tisch.comp-genomics.org). For the HCC patient group, we used the Smart-seq2 data published by Zhang 21. We analyzed these cells in parallel with the R packages Seurat (version 3.1.0) 22 and SingleR (version 1.2.4). The feature barcode matrixes were imported using Read10X and CreadSeuratObject. For all groups, genes that were expressed in a minimum of 5 cells were applied, while cells with greater than 15% mitochondrial genes and fewer than 250 or more than 6,000 unique-molecular-identifier (UMI) counts were removed, which generally indicates poor-quality cells and sequencing errors. In total, we obtained 115,737 PBMCs from the two healthy datasets and 2,024 HCC PBMCs from the HCC dataset. The remaining cells were used for downstream analysis along with the patient object. After global log normalization and scaling to 10,000 transcripts per cell using NormalizeData, variable genes in the control and patient objects were identified separately by the command FindVariableFeatures, and cell types were identified by the SingleR command.

Combined SingleR (v1.8.1) 23 package and markers from documents, we identified the final cell types. We operated CellphoneDB (v3.1.0) 24 on HCC PB expression matrix and health PB respectively to investigate cell-cell interactions, by usage parameter 'cellphonedb method statistical analysis HCC_cell.txt HCC_count.txt --output-path=. /HCC_out'. Here we change gene symbol to gene ensemble ID matched with CellphoneDB by biomaRt 25. To figure out the intensity of interaction and potential ligand-receptor pairs, we use 'cellphonedb plot heatmap plot' command and ggplot2 R package to draw heatmaps and dot diagrams.

### Patients and Healthy Controls

We recruited a total of 89 patients diagnosed with HCC and 33 healthy volunteers for this research during 2016-2019. All patients were confirmed by biopsy, and none of them had received surgery or TACE before blood sample collection. No patients had any other tumors, blood diseases, autoimmune diseases, or inflammatory conditions. Among them, data of 15 HCC patients who underwent liver resection were collected and compared with their preoperative data. Healthy controls were matched for age and sex.

### Isolation of Peripheral Blood Mononuclear Cells (PBMCs)

Fresh heparinized venous blood samples were obtained from 122 subjects before treatment and were separated immediately. PBMCs were isolated by standard Ficoll-Paque Plus (GE Healthcare, 17-1440-02) density gradient centrifugation. The viability of PBMCs was evaluated by trypan blue dye staining. And we used an adjusted concentration of 1×10^6^/ml PBMCs in each tube for subsequent operation.

### Cell Staining and Flow Cytometry Analysis

We used monoclonal antibodies against CD19 (APC-Cy7), CD1d (APC), CD5 (FITC), CD21 (PE-Cy7), and CD23 (PerCP-Cy5.5) from BD Bioscience for cell staining. PBMCs were surface stained with these monoclonal antibodies against human antigens in tubes according to the manufacturer's instructions. All samples were incubated at room temperature without exposure to light for 20 mins. Flow cytometry was performed using a BD FACS Canto II to examine the percentages of B lymphocyte subsets. A total of 1×10^6^ cells were collected, and data were analyzed using FlowJo 10. We showed our gating strategies in **Figure 3**. Lymphocytes were first acquired in the FSC-A/SSC-A lymphocyte gate, and then subsets of B cells were identified based on the following phenotypes: B10 cells (CD19^+^CD5^+^CD1d^high^), MZB cells (CD19^+^CD21^+^CD23^-^), and FoB cells (CD19^+^CD21^-^CD23^+^) 15.

### IL-10 Detection

We used commercially available human enzyme-linked immunosorbent assay (ELISA) kits (Lianke bio, 70-EK110/2-96) to quantify the concentration of the serum cytokine IL-10. We ran tests on all samples in duplicate. All tests were performed in accordance with the manufacturer's protocols.

### Statistical Analysis

All statistical analyses were performed using Graphpad prism software (version 6.01). Group differences were evaluated using a two-tailed Student's *t*-test, and statistical significance was defined as a two-sided P value <0.05. Pearson correlations were used to estimate the association between the two parameters.

### Graphical model figure

The Graphical model figure was accessed by Figdraw.

## Results

### B Lymphocyte Subsets lineage in HCC patients identified by Single-cell RNA-seq data analyzation

**Figure 1:**
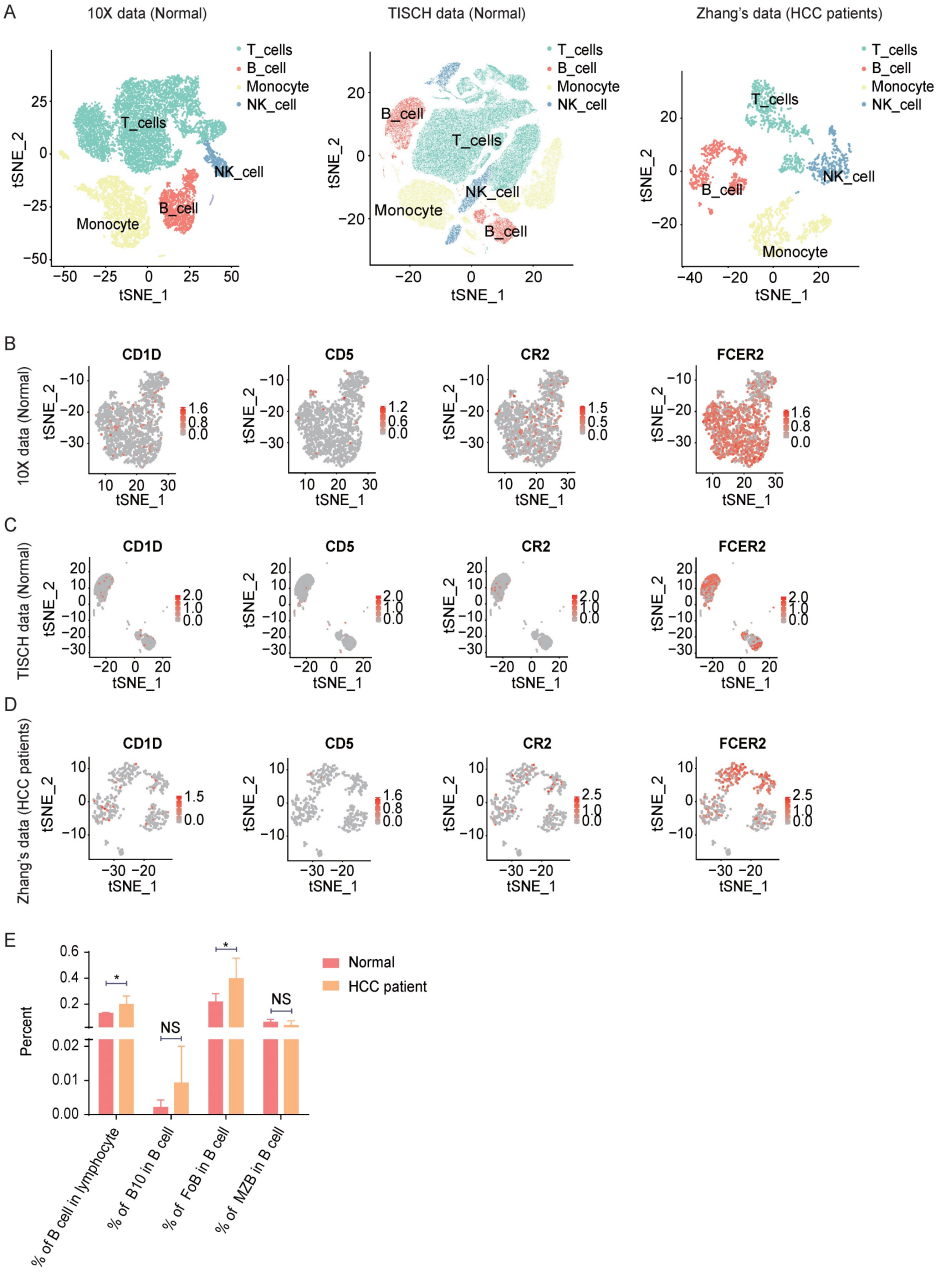
** Analyzation of Lymphocyte subsets in HCC PBMC with public scRNA-seq data.** (A) tSNE ploting showed the cell clusters in Normal and HCC PBMC. (B-D) The expression of markers in B cell or B cell subsets. (E) The B cell or B cell percentage sets in normal and HCC patients. *P < 0.05.

To investigate the function of B lymphocytes in hepatocellular carcinoma development, firstly, we analyzed seven public healthy PBMC single-cell datasets from 10X Genomics and TISCH. For the HCC patient group, we used Zhang's 21 Smart-seq2 data. In total, we obtained 115,737 healthy PBMCs from the seven datasets and 2024 HCC PBMCs from six patients **(Figure 1A and Figure S1A)**. Paired with flow cytometry markers, CD19 (CD19), CD1D (CD1d), CD5 (CD5), CR2 (CD21), and FCER2 (CD23) were used to filter count matrixes **(Figure 1B-D and Figure S1B)**. We discovered that B cells (CD19^+^) were more abundant in the HCC PBMC datasets than in the healthy datasets **(Figure 1E)**. As a percentage of total B cells, B10 cells (CD1D^+^, CD5^+^) were also more abundant in HCC, but the difference was not significant **(Figure 1E)**. FoB cells (FCER2^+^, CR2^-^) in HCC PBMCs were significantly more abundant than those in healthy PBMCs **(Figure 1E)**, while the percentage of MZB cells (CR2^+^, FCER2^-^) in HCC PBMCs was indistinctly less than that in healthy PBMCs **(Figure 1E)**. These analyzations indicate B Lymphocyte Subsets in HCC Patients Changed.

### Identification of DEGs and possible ligand-receptor interactions between B cell subsets and other lymphocytes

**Figure 2:**
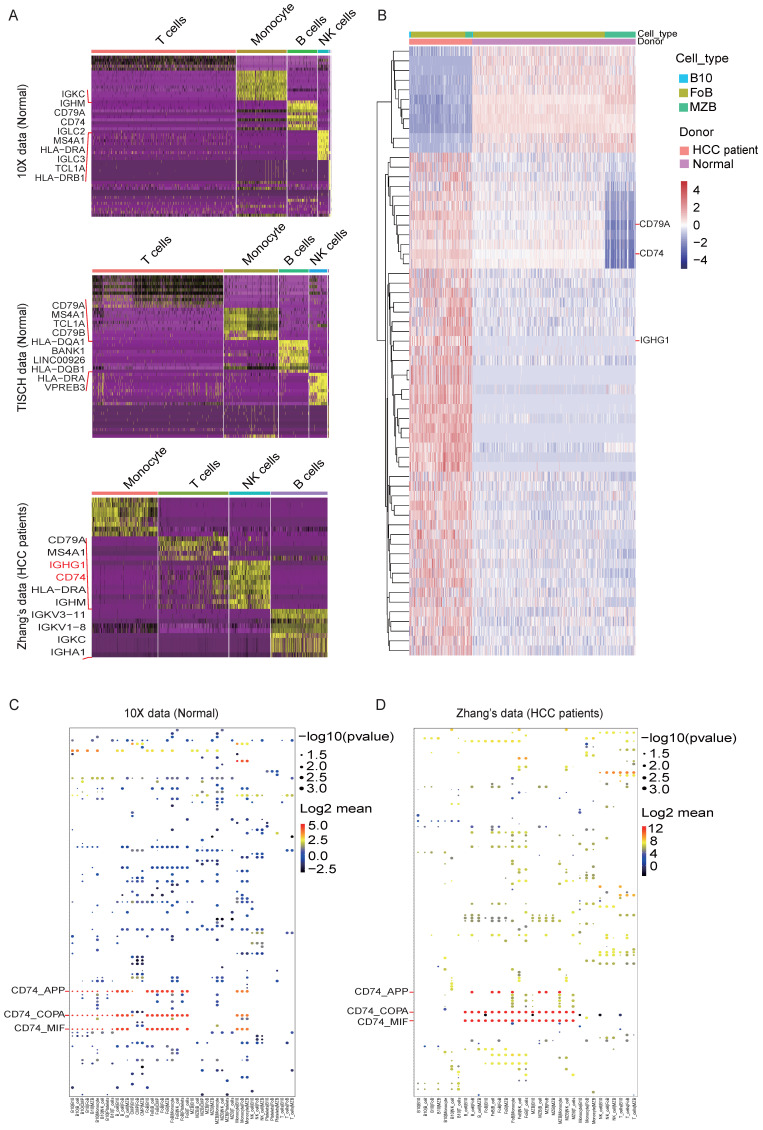
** DEGs and possible ligand-receptor interactions between different cell types analysis.** (A) Marker genes of lymphocyte subsets in the three datasets. (B) Differentially expressed genes (DEGs) of PBMC between HCC and healthy donors are analyzed by R packages DESeq2 (v1.16.1). DESeq2 provides an acceptable strategy to determine the difference in a single cell dataset. We use p adjust < 0.01 and log2fold-change > 0.3 (or < -0.3) as cutoff to interpret significant differentially expressed genes to plot heatmap. Here we showed the different express genes of Normal group datasets from 10X and HCC group datasets from Zhang. (C-D) Cell-cell interactions are operated by CellPhoneDB, and ggplot2 was used for drawing dot diagrams. The possible ligand-receptor interactions between different cell types in the Normal group (C) and the HCC group (D) manifested decreased CD74-related cell-cell interaction in HCC.

We defined the character of these cell clusters with differential expression analysis, and the heatmap of the top 10 most differentially expressed markers were shown in** Figure. 2A**, here we found out that *IGHG1* and *CD74* were enhanced expressed in the B cell cluster in the HCC dataset compared with the other two Normal datasets. The comprehensive and detailed cell-lineage-specific marker genes of different lymphocyte cells were displayed in **Table S1**.

Subsequently, we further analyzed the DEGs in B cell subsets. Consistent with the DEGs in the B cell cluster, *IGHG1* and *CD74* were dramatically high expressed in B10 cell and FoB cell in HCC compared with Normal (**Figure. 2B**). The full list of DEGs were displayed in **Table S2**.

To explore the interactions network of B cell subsets with other lymphocytes in HCC, we performed the ligand-receptor analysis in the Normal dataset and HCC dataset respectively. Firstly, we operate ssGSEA on different B cells in different histology by GSVA (v1.42) R package 26. We find a mass of gene sets changed in B cells between HCC and health. Here we focus on biological functions and GO_BP in Hallmark 27. We find B cell related function are obviously repressed in HCC B cells globally (**Figure S1C**). Target B subclasses (B10/MZB/FoB) have differential biological process. Healthy MZBs are involved in B cell proliferation; B10s have highly expressed inflammatory response; FoBs have strong B cell receptor signal (**Figure S1D**).

We focus on ligand-receptor pairs found in B10, FoB, MZB, and other B cells, compared with landscape of interaction in health blood, MZB and B10 have decreased interaction with monocytes (**Figure S1E & F**). For details, the dot diagrams manifested CD74-related cell-cell interaction were decreased in HCC (**Figure. 2C and 2D**), which implied the potential function mechanism of B cell subsets in HCC development. The total list of potential cell-cell interactions was displayed in **Table S3**.

### Flow cytometry analysis revealed increased B10 cells and decreased MZB cells in peripheral blood of HCC patients compared with Healthy Controls

To confirm the changes in B cell subsets in HCC, we recruited a cohort of 89 patients diagnosed with HCC and 33 healthy volunteers to further study this phenomenon.

Firstly, the Liver function of all the subjects was evaluated with tests of venous blood samples for total protein (TP), albumin (ALB), glutamine transaminase (γ-GT), glutamyl-pyruvic transaminase (ALT), alkaline phosphatase (ALP), direct bilirubin (DB), total bilirubin (TB) and alpha-fetoprotein (AFP). Clinical characteristics are expressed as the mean ± standard error (SE) or number (**Table 1**).

**Figure 3:**
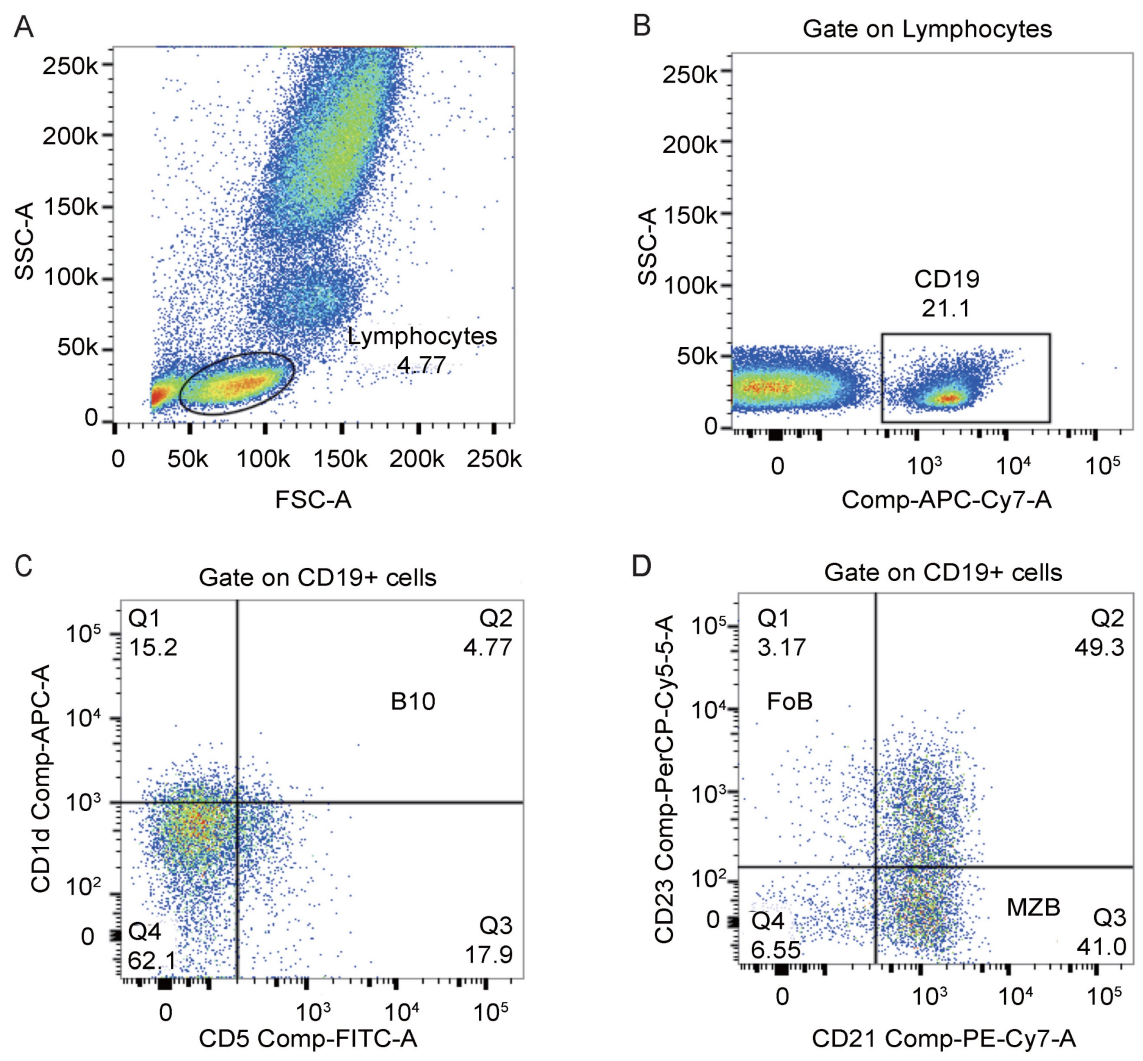
** Flow cytometry analysis of the percentage of B lymphocyte subsets in peripheral blood.** (A) Lymphocytes were defined on the FSC-A/SSC-A gate. (B) CD19^+^ cells were gated on lymphocytes. (C) The dot plot represented the gating strategy on B10 cells derived from CD19^+^ cells. (D) The dot plot represented the gating strategy on MZB cells and FoB cells derived from CD19^+^ cells.

**Figure 4:**
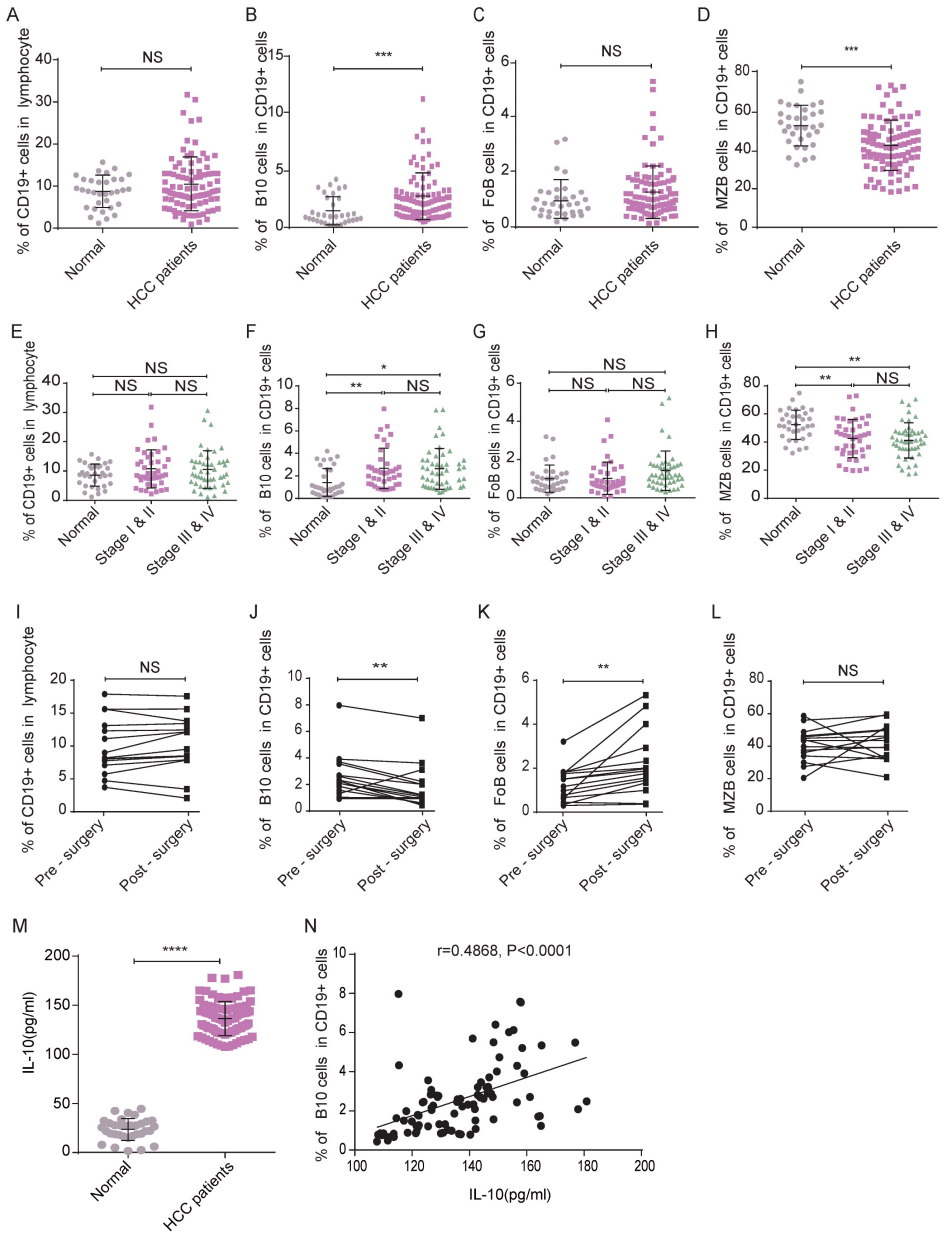
** Changed frequency of B10 cells and associated IL-10 concentration may function positively in HCC.** (A-D) Frequencies of circulating B lymphocyte subsets were collected and analyzed, compared with healthy subjects, there was no difference in the frequencies of CD19^+^ B cells (A) and FoB (C) cells, but the frequency of B10 cells (B) was increased while the frequency of MZB cells (D) was decreased. (E-H) The frequencies of CD19^+^ B cells and their subsets (E-H) in different clinical stages. (I-L) Perioperative Changes of CD19^+^ B cells and its subsets after surgery. (M) The serum concentration of IL-10 in HCC patients was significantly higher compared with healthy controls. (N) Scatter diagram of concentration of IL-10 and frequency of B10 cells. *P < 0.05, **P < 0.01, *** P < 0.001, **** P < 0.0001.

Secondly, following the gating strategies shown in **Figure 3**, we detected the frequencies of B lymphocyte subsets in HCC patients and healthy controls by flow cytometry analysis. Although there was no significant difference between the two groups of participants in the frequency of CD19^+^ B lymphocytes (**Figure 4A**), the frequency of B10 cells in HCC patients was significantly higher than that in healthy donors (**Figure 4B**), which indicated that B10 cells may play a key role in HCC progress.

We also measured the percentage of FoB cells and found no significant difference between the two groups (**Figure 4C**). MZB cells were less frequent in HCC patients than in healthy controls (**Figure 4D**). The trends in B lymphocyte subset frequencies remained the same after we adjusted for age and sex (data not shown).

### B10 cell was associated with the early TNM stage and Perioperative

To verify the effects of tumor progression on changes in B cell subsets, we analyzed patients' clinical stages. Here, there were 31 HCC patients in stage I, 10 in stage II, 13 in stage III and 35 in stage IV. We separated the patients into two groups according to their stages. Group A consisted of patients with stage I-II disease. Group B consisted of patients with stage III-IV disease. As shown in **Figure 4**, compared with that in the healthy control group, the frequencies of B10 cells in groups A&B were higher (**Figure 4F**), and those of MZB cells in groups A&B were lower (**Figure 4H**). However, we did not observe a significant difference in the frequency of B10 cells or MZB cells between groups A and B, which indicated that the changes in B cell subsets might occur at an early stage. The frequency of CD19^+^ cells (**Figure 4E**) and FoB cells (**Figure 4G**) in group A was not significantly different from that in the healthy group or group B.

To validate whether the function of surgery on the frequency of B cell subsets, we analyzed samples from 15 HCC patients who underwent liver resection. Compared with the frequency of B10 cells in the 15 patients before surgery, that in the HCC patients five days after surgery was decreased (**Figure 4J**). We observed a significant elevation in the percentage of FoB cells in the HCC patients five days after surgery (**Figure 4K**). The percentage of CD19^+^ B cells (**Figure 4I**) and MZB cells (**Figure 4L**) in the post-surgery patients was not different from the pre-surgery level. These results indicated that the changed B10 cell in HCC patients may affect the development of this disease.

### Elevated IL-10 concentration might enhance the development of HCC

Since B10 cells mainly secrete IL-10 and perform immune suppress function, we furtherly examined the serum concentration of IL-10, which was significantly increased in HCC patients compared with healthy controls (**Figure 4M**). In addition, there was a positive correlation between the IL-10 concentration and B10 frequency in HCC patients (r=0.4868, *P*<0.0001, **Figure 4N**). These results indicated that B 10 cells and IL-10 might be the positive functional molecules in HCC development.

As B10 markers showed (CD5^+^, CD1D^+^) at single-cell mRNA level, B10 cells are not identified on a large scale, along with low expressed IL10. We find IL10 expressed higher in B10 cells than in other B cells, though not significant enough due to the limited sample size (**Figure S1G**). We operate survival analysis in TCGA LIHC project, total 364 patients with clinical information, and figure KM-curve, dividing the cohort into IL10-high and IL10-low groups (**Figure S1H**). Disappointingly, expression of IL10 has no significant evidence correlated with prognostic survival.

## Discussion

Recent studies have shown that the tumor microenvironment plays an essential role in immunopathogenesis and the establishment of primary hepatocellular carcinoma 28, 29. Many studies have highlighted the importance of T cell subsets, such as Treg and Th17 cells, in immune tolerance functions in HCC patients 30. B lymphocytes also greatly contribute to regulating the immune response and maintaining immune homeostasis due to their capabilities to present antigens, produce cytokines, and activate effector T cells 31, 32. However, the roles of B cell subsets in regulating the immune response in HCC patients remain unclear.

The current research is the first study to explore the frequencies of different B cell subsets in the peripheral blood of HCC patients. B cell subsets are identified according to distinct surface phenotypes and different immunological functions in the antitumor response 33.

The frequency of B10 cells was elevated in the peripheral blood of HCC patients compared with that of healthy controls. As regulatory B cells, B10 cells are capable of suppressing immune activity in human and mouse models 34. This result is by the findings of other reported studies, which suggest an increased Breg cell frequency in patients with liver tumors 35. B10 cells can facilitate the expansion of Treg cells via transforming growth factor-β 36. Tumor immune function and cell mechanisms in HCC have yet to be studied. B10 cells are also the main source of IL-10, a product of B cells. We examined the concentration of IL-10 in the serum of our subjects and found that the serum IL-10 concentration in HCC patients was higher than that in healthy controls. The B10 cell frequency and IL-10 concentration showed a positive correlation. To our knowledge, IL-10 can be produced by other cells, including Treg cells, and can be regulated by various pathways 37. Thus, more *in vivo* research should be done to explore this correlation. IL-10 produced by B10 cells can participate in immune response suppression by targeting Treg cells in the tumor site 31.

An interesting finding was the lack of a significant change in the frequency of FoB cells between HCC patients and healthy controls, while the frequency of MZB cells was pronouncedly decreased in the peripheral blood of patients. However, the condition that instructs transitional B cells to differentiate into MZB or FoB cells remains unclear. The differentiation and expansion of B cell subsets are triggered by BCR signaling and the Notch pathway 16. Both MZB cells and FoB cells have strong antitumor potential since they can participate in positive regulation of the immune response by activating naïve CD4^+^ T cells and displaying pathogens in the peripheral blood 38. The decreased frequency of MZB cells may indicate that tumor cells have already escaped tumor immune surveillance. As reported, FoB cells are weaker and slower than MZB cells in activating T cells in the immune response 14. This might be a reason why the frequency of FoB cells remained unchanged.

Our results also showed that the frequency of B10 cells and MZB cells changed from stages I&II and remained relatively constant across different stages. The reason is still unclear, but it could reflect the function of B10 cells or MZB cells in HCC prognosis. This result indicated that these cells might be associated with a more advanced HCC tumor stage and that the B10 or MZB cell-induced antitumor response might still exist in advanced-stage HCC, though it is not enough for preventing tumor cell escape.

Fifteen patients received surgical treatment, and we examined the frequencies of B cell subsets 5 days after each patient underwent surgery. Interestingly, we found that the frequency of B10 cells tended to decrease after surgery and that the FoB cell frequency exhibited a pronounced increase after surgery. The change in B10 cells was consistent with that in other studies investigating tumor prediction and prognosis. As reported previously, B cells infiltrate liver tumors, where they acquire an immunosuppressive phenotype and convert into Breg cells, including B10 cells, which can suppress the antitumor immune response in tumor-bearing patients. The frequency of B10 cells decreased after tumor resection, and the immune response recovered to some extent. It is reasonable that the frequency of FoB cells increases when patients undergo resection. Patients with different immune statuses should be treated in different ways, and these B cells might be potential targets in the treatment of HCC patients.

## Supplementary Material

Supplementary figure and table legends.

Supplementary table 1.

Supplementary table 2.

Supplementary table 3.

## Figures and Tables

**Table 1:** Participants' characteristics at baseline.

Group	Healthy control	HCC patients	p-value
Male (%)	78.79	77.53	
Age (years)	54.54±2.08	55.44±1.2	0.72
AFP (ng/ml)	3.7±0.4	1129±156.9	<0.0001^****^
CEA (ng/ml)	1.92±0.24	16.8±6.5	0.25
TB(μmol/l)	13.5±0.9	18.9±1.2	0.02^*^
DB(μmol/l)	2.82±0.23	10.26±0.94	<0.001^***^
ALP(U/L)	74.1±4.2	134.9±11.4	0.02^*^
ALT(U/L)	19.6±2.7	83.7±19	0.08
GGT(U/L)	36.58±7.71	152.43±18.93	0.001^***^
TP(g/l)	71.4±4.2	70.4±0.6	0.4
ALB(g/l)	46.35±0.43	40.45±0.65	0.01^**^
A/G	1.85±0.04	1.44±0.04	0.0001^****^

Values of liver function were described by mean ± SD. *, **, *** and **** stand for P < 0.05, 0.01, 0.001 and 0.0001 respectively.

## Abbreviations

HCC: hepatocellular carcinoma
B10 cell: interleukin-10-producing regulatory B cell
MZB cell: marginal zone B cell
FoB cell: follicular B cell
IL-10: interleukin-10
HBV: hepatitis B virus
HCV: hepatitis C virus
TACE: transarterial chemoembolization
Treg cell: regulatory T cell
Th17: T helper 17
UMI: unique-molecular-identifier
TP: total protein
ALB: albumin
γ-GT: glutamine transaminase
ALT: glutamyl-pyruvic transaminase
ALP: alkaline phosphatase
DB: direct bilirubin
TB: total bilirubin
AFP: Alpha-fetoprotein
ELISA: enzyme-linked immunosorbent assay
