# Supplementary material for: Aberrant alteration of peripheral B lymphocyte subsets in hepatocellular carcinoma patients
Source: Int J Med Sci. 2023 Jan 22;20(2):267–77. doi: 10.7150/ijms.79305 (PMC9925981; doi:10.7150/ijms.79305)
Supplement: Supplementary file 1 — Supplementary figure and table legends. [file ijmsv20p0267s1.pdf]

Figure S1. Analyzation of B cell subsets and IL-10 expression in HCC PBMC with public scRNA-seq data. (A) tSNE ploting showed the B cell clusters in Normal and HCC PBMC. (B) The expression of markers in B cell subsets. (C) ssGSEA analyzation on different B cells in different histology, B cell related hallmark biological functions and GO_BP are obviously repressed in HCC B cells globally. (D) Biological process of target B subclasses (B10/MZB/FoB) were also analyzed by ssGSEA. (E-F) The landscape of interaction of B cell in both health and HCC PB. (G) Expression of IL-10 in B10 cell and other B cell subtypes at single-cell mRNA level. (H) Survival analysis in TCGA LIHC project, total 364 patients with clinical information, and figure KM-curve, dividing the cohort into IL10-high and IL10-low groups.

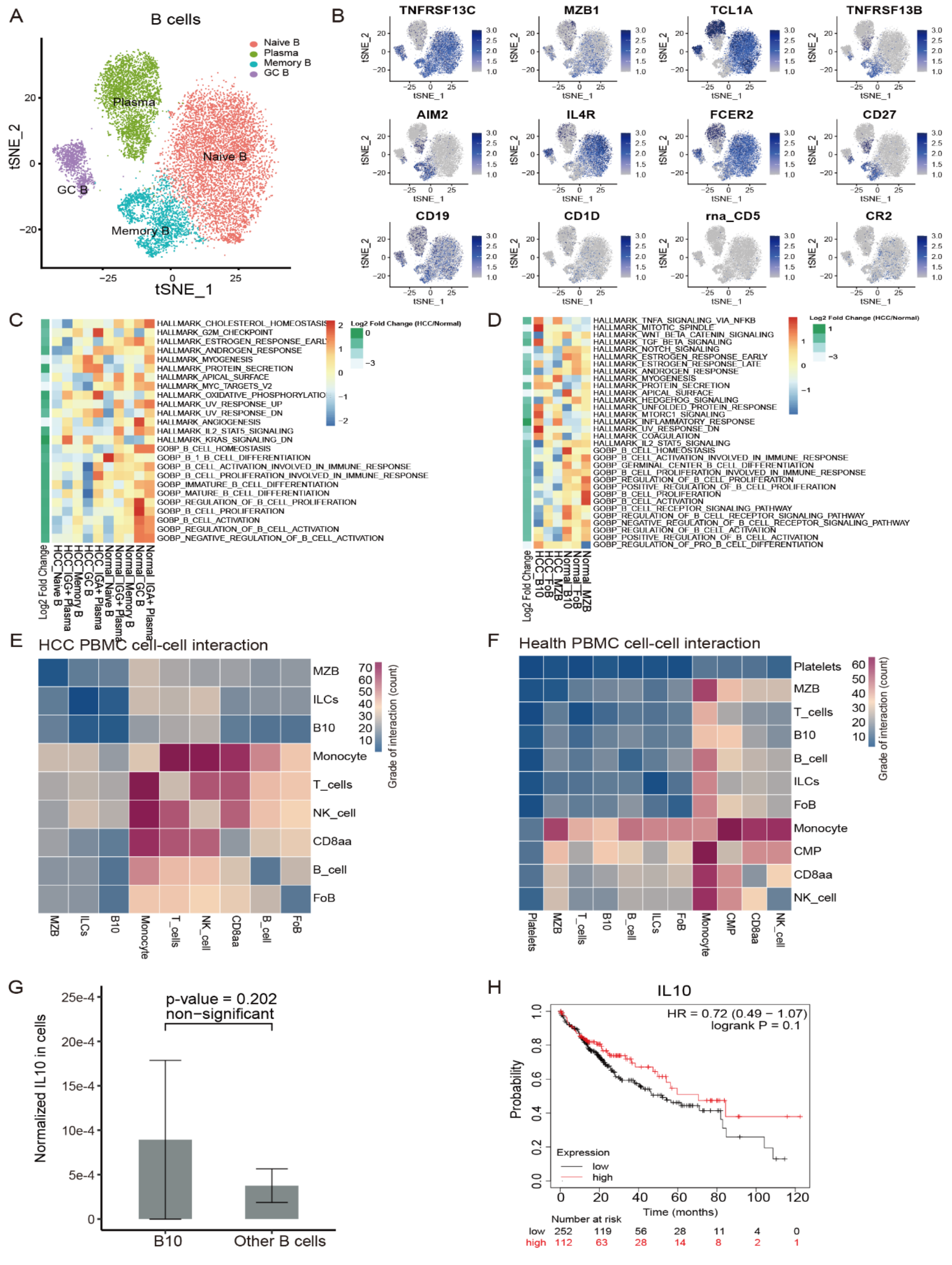

Figure S1

Table S1. Marker Genes by Cell.

Marker genes of lymphocyte subsets in the three datasets.

Table S2. DEG_10x_vs_zhang's data.

Differentially expressed genes (DEGs) of PBMC between HCC and healthy donors are analyzed by R packages DESeq2 (v1.16.1). DESeq2 provides an acceptable strategy to determine the differences in single-cell datasets. We use p adjust < 0.01 and log2fold-change > 0.3 (or < -0.3) as a cutoff to interpret significant differentially expressed genes to plot heatmap.

Table S3. Cell interaction.

Cell-cell interactions are operated by CellPhoneDB, and ggplot2 was used for drawing dot diagrams.
